# Fix or Adapt? Rethinking the Management of Complex Autonomic Dysfunction in an Older Adult Following Orthopedic Surgery

**DOI:** 10.1111/ggi.70237

**Published:** 2025-10-28

**Authors:** Calvin D. De Louche, Sudarshanie Palliyage

**Affiliations:** ^1^ Department of Orthogeriatric Medicine Ealing Hospital, London North West University Healthcare NHS Trust London UK; ^2^ Faculty of Medicine, Department of Surgery and Cancer Imperial College London London UK

To the Editor,

1

We report the case of a man in his late sixties who presented following a syncopal fall resulting in a left neck of femur fracture. The patient underwent surgical fixation with a long gamma nail, which was completed without complication. The patient's past medical history was significant for atrial fibrillation, type 2 diabetes mellitus (T2DM) complicated by diabetic maculopathy/proliferative retinopathy, hypertension, and stage 3 chronic kidney disease (CKD), baseline creatinine ~112 μmol/L; eGFR ~59 mL/min/1.73 m^2^.

Following surgery, the patient underwent rehabilitation but exhibited symptomatic significant orthostatic hypotension (OH) on standing with systolic blood pressure (BP) drops ≥ 40 mmHg (Table 1). Daytime supine BP ranged from 160/90 to 210/100 mmHg. Findings were consistent with severe supine hypertension (SH) and marked OH. Subsequent autonomic function testing revealed mixed vagal and sympathetic autonomic dysfunction.

**TABLE 1:** Therapeutic strategies, non‐pharmacological interventions, blood pressure fluctuations, and renal function during admission.

Category	Intervention/Finding	Outcome/Reason for cessation	Notes/Clinical impact
Pharmacological management	Antihypertensives (losartan, nifedipine)	Exacerbated postural symptoms	Contraindicated due to worsening OH
	Midodrine, droxidopa	Contraindicated in CKD	Use advised with caution; not trialed
	Fludrocortisone	Precipitated acute renal failure	Required ITU admission and hemofiltration
	Pyridostigmine, octreotide	Not trialed	Safety profile in CKD uncertain; not on local formulary
Non‐pharmacological interventions	Head‐up tilt positioning, fluid optimization, compression garments (including abdominal binders)	Ineffective	No improvement in symptomatic OH
Blood pressure fluctuations	Magnitude of postural BP drops (systolic/diastolic, mmHg): −67/−27, −17/−13, −52/−29, −71/−30, −47/−26	Symptomatic with dizziness and light‐headedness	Severe and unpredictable BP variability prevented safe mobilization. The patient was unable to tolerate standing > 1–2 min, restricting physiotherapy to brief, assisted attempts and effectively halting structured rehabilitation.
Renal function trajectory	Baseline: Creatinine 112 μmol/L; eGFR 59	Stable	—
	Post‐fludrocortisone: Creatinine 459 μmol/L; eGFR 11	Acute kidney injury	ITU admission, hemofiltration initiated
	Subsequent values in time order: Cr 411 μmol/L (eGFR 12) Cr 380 μmol/L (eGFR 13) Cr 364 μmol/L (eGFR 14) Cr 330 μmol/L (eGFR 16) Cr 300 μmol/L (eGFR 18) Cr 283 μmol/L (eGFR 19) Cr 276 μmol/L (eGFR 20) Cr 257 μmol/L (eGFR 21) Cr 200 μmol/L (eGFR 29) Cr 184 μmol/L (eGFR 32) Renal function had not returned to baseline at discharge	Gradual improvement	Recovery trajectory following hemofiltration

Abbreviations: BP, blood pressure; CKD, chronic kidney disease; Cr, creatinine; eGFR, estimated glomerular filtration rate; ITU, intensive treatment unit; OH, orthostatic hypotension; SH, supine hypertension; T2DM, type 2 diabetes mellitus.

This posed significant management challenges and severely limited his ability to participate in rehabilitation as he was unable to maintain upright posture due to refractory symptomatic OH. While non‐pharmacological measures, including fluid optimization, compression garments, and a head‐up sleeping position were all ineffective, the distinctive challenge of this case was that his advanced CKD severely constrained pharmacological treatment options, underscoring the critical need to prioritize alternative rehabilitation strategies.

This case highlights the challenges of managing autonomic dysfunction and its implications for rehabilitation following orthopedic surgery in older adults. The co‐existence of SH and OH posed a significant therapeutic dilemma, particularly when treatment for one condition risked exacerbating the other.

Autonomic dysfunction, the failure of components of the autonomic nervous system to function properly, may result from autoimmune disease, neurodegenerative pathology, infection, neoplasia, or toxins [1]. Metabolic diseases like diabetes mellitus are also a leading cause of acquired autonomic dysfunction [2]. For this patient, autonomic testing confirmed mixed sympathetic and parasympathetic involvement, consistent with diabetic autonomic neuropathy.

OH, defined as a sustained drop of ≥ 20 mmHg systolic or ≥ 10 mmHg diastolic BP within 3 min of standing, is a common presentation in older adults [3] and may be caused by volume depletion, cardiac dysfunction or adverse medication reactions [3]. SH, characterized by significant hypertension on lying down, is also frequently observed in older adults [2]. Both OH and SH are characteristic manifestations of autonomic dysfunction, particularly baroreflex failure, and result in marked BP variability [4].

The treatment of this condition requires a delicate balance of therapies. Management of SH typically involves using antihypertensive medications [4]. In this case, attempts to manage SH using losartan and nifedipine exacerbated postural symptoms. For OH, first‐line management focuses on non‐pharmacological strategies such as head‐up tilt positioning, fluid optimization, and compression garments [5]; these were all trialed in this patient with little success. Pharmacological options such as fludrocortisone, midodrine, and droxidopa are frequently used, but can be particularly difficult to manage in renal impairment, due to altered drug handling and response [6]. This was evident in our patient whose renal function markedly deteriorated following fludrocortisone administration, ultimately necessitating hemofiltration (Table 1). Alternative agents such as pyridostigmine, an acetylcholinesterase inhibitor, and octreotide, a somatostatin analogue, have shown benefit in patients with autonomic failure and OH [7, 8]. However, their safety profile in advanced CKD remains incompletely understood, and warrants further evaluation before routine use.

Autonomic failure presents a major challenge to rehabilitation in older adults. Hip fracture is a leading cause of hospitalization, and early mobilization is critical in reducing morbidity and mortality [9]. In many cases, older adults achieve some degree of functional recovery within the first few months post‐surgery, but regular therapy is required to maximize recovery outcomes [10]. Optimizing BP in autonomic dysfunction requires a delicate balance: low enough to reduce the risks of SH and its cardiovascular sequelae, yet high enough to permit safe engagement in rehabilitation.

For this patient, the inability to tolerate even brief upright posture severely curtailed physiotherapy efforts and prolonged the admission. Therefore, early recognition of when pharmacological management is unlikely to be effective enables clinicians to transition to supportive strategies aimed at adaptation rather than disease modification. This approach should include detailed patient education, individualized BP targets to mitigate postural drops, and the use of adaptive mobility aids to further support the patient (e.g., wheelchair for mobilizing, frames for stability on standing). Timely transition to an adaptive care plan may be essential to facilitating rehabilitation and improving patient outcomes.

In older adults with severe autonomic dysfunction and CKD, pharmacological therapy may carry more risks than benefits, and early prioritization of adaptive rehabilitation strategies should be considered.

## Author Contributions

Calvin D. De Louche: involved in the day‐to‐day care of the patient. Wrote and edited the manuscript and provided comments on the final version. Sudarshanie Palliyage: involved in the day‐to‐day care of the patient. Reviewed and edited the manuscript and approved the final version.

## Consent

Written informed consent was obtained from the patient for publication of this case report.

## Conflicts of Interest

The authors declare no conflicts of interest.

## Data Availability

Data sharing not applicable to this article as no datasets were generated or analyzed during the current study.
